# A Mathematical Model of Regenerative Axon Growing along Glial Scar after Spinal Cord Injury

**DOI:** 10.1155/2016/3030454

**Published:** 2016-05-04

**Authors:** Xuning Chen, Weiping Zhu

**Affiliations:** Shanghai Institute of Applied Mathematics and Mechanics, Shanghai University, Shanghai 200072, China

## Abstract

A major factor in the failure of central nervous system (CNS) axon regeneration is the formation of glial scar after the injury of CNS. Glial scar generates a dense barrier which the regenerative axons cannot easily pass through or by. In this paper, a mathematical model was established to explore how the regenerative axons grow along the surface of glial scar or bypass the glial scar. This mathematical model was constructed based on the spinal cord injury (SCI) repair experiments by transplanting Schwann cells as bridge over the glial scar. The Lattice Boltzmann Method (LBM) was used in this model for three-dimensional numerical simulation. The advantage of this model is that it provides a parallel and easily implemented algorithm and has the capability of handling complicated boundaries. Using the simulated data, two significant conclusions were made in this study: (1) the levels of inhibitory factors on the surface of the glial scar are the main factors affecting axon elongation and (2) when the inhibitory factor levels on the surface of the glial scar remain constant, the longitudinal size of the glial scar has greater influence on the average rate of axon growth than the transverse size. These results will provide theoretical guidance and reference for researchers to design efficient experiments.

## 1. Introduction

Spinal cord injury (SCI) is the damage to the spinal cord that results in a loss of function such as mobility or feeling. An injured spinal cord has a poor intrinsic capacity for regeneration, although some functional recovery does occur. The failure to regenerate is caused by a combination of factors, including neuroinflammation, axonal disruption, death of neurons, glial scar formation, the release of myelin-associated inhibitory molecules, and the lack of growth promoting molecules. It is clear that effective treatment will require a multifaceted combination of strategies. A lot of effort has been made to promote CNS regeneration but with only limited success [1–6].

Trauma or disease of a nerve in a mature mammal may result in a massive multiplication of glial cells around the damaged region, which eventually form a dense scar. This glial scar plays a dual role as chemical and mechanical barriers to the axonal regeneration of injured neurons [7–10]. Many chemical factors from glial scars have been identified, including Nogo-A, Nogo-B, Nogo-C, myelin-associated glycoprotein, and oligodendrocyte-myelin glycoprotein, to inhibit axonal growth [11–13]. On the other hand, the size, shape, and hardness of the glial scars represent the mechanical aspects that resist the extension of the regenerative axons.

Some experimental strategies have been employed to improve the CNS regeneration. These strategies include (1) complementing favorable promoting factors in CNS (such as nerve growth factor (NGF)) [14]; (2) decreasing expression of the inhibiting factors such as Nogo-neutralizing antibody (IN-1) and chondroitinase ABC (Chase-ABC) [15, 16]; and (3) repairing or reconstructing myelin. For example, transplant of Schwann cell into the SCI site provides a permissive cellular substrate which may enable axons to pass through the scar area and find their targets [17]. However, experimental studies to examine many factors simultaneously are often time-consuming, costly, and laborious. The optimal concentration ratios of various factors for growth of regenerating axons in a suitable microenvironment are valuable information for researchers in experimental design. The technologies of mathematical modeling could provide an opportunity to elucidate the ratio and distribution law of various impact factors.

Several mathematical models have been developed to describe axonal growth [18–25]. These models generally consist of two parts: (1) the reaction-diffusion equation describes the transmission of nerve factors and other guidance molecules during development and (2) the axonal “growth equation” (based on the cell chemotaxis principle) describes the growth of axons determined by the concentration gradient of the guidance molecules. By allowing for noise in axonal guidance cues and randomized changes in axon growth substrates, a stochastic component has been included in the growth equation [19]. A previous study showed a two-dimensional finite difference in the solution and calculation program of “parabolic equations with a gradient term” [23]. Another study obtained a large-scale, two-dimensional simulation result using parallel computing [20]. One of our previous studies used a three-dimensional finite difference method and reported simulation result [22]. The Lattice Boltzmann Method (LBM), a numerical method commonly used for fluid simulation [26–29], directly describes problems and is convenient for implementing parallel computing. Nevertheless, these studies did not consider regenerating axonal growth in a deprived environment.

**Figure 1:**
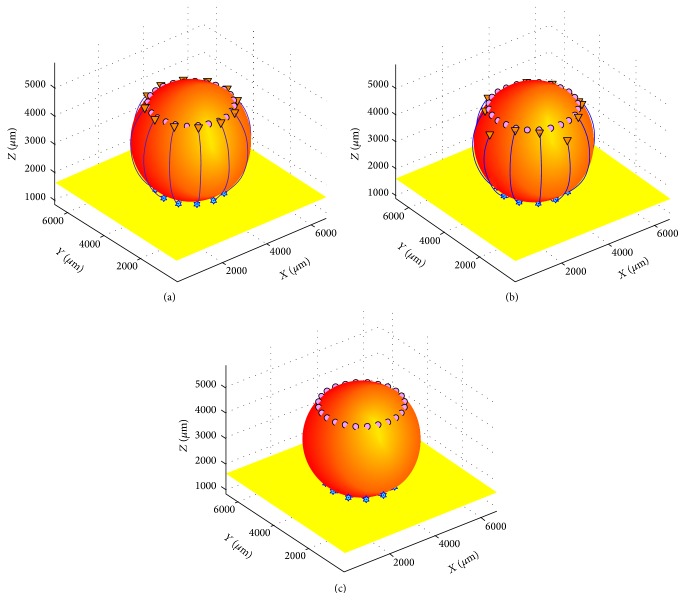
The effects of the release rate of growth inhibitory factors on regenerating axons growing along glial scar (*η*_2_ = 3%, 5%, and 7%). (a) *η*_2_ = 3%; *r*_*a*_ = *r*_*z*_ = 0.26 × *L*. When the release rate of growth inhibitory factors was relatively low (*η*_2_ = 3%), regenerating axons could successfully navigate across the glial scar and connect to their target cells with the support of Schwann cells and the guidance of NTFs concentration gradients of the target cells. (b) *η*_2_ = 5%; *r*_*a*_ = *r*_*z*_ = 0.26 × *L*. When the release rate of growth inhibitory factors was relatively high (*η*_2_ = 5%), some axons could grow successfully to reestablish connections with their target cells, whereas others stopped growing when they reached halfway. (c) *η*_2_ = 7%; *r*_*a*_ = *r*_*z*_ = 0.26 × *L*. All regenerating axons immediately stopped growing when they encountered the glial scar when the release rate of growth inhibitory factors was over 7%.

In this study a numerical simulation method, which was based on the experiment for the SCI repair by Schwann cell transplantation [17], was used to explore the regenerative axons growing along the glial scar surface. In this model, glial scar was simplified to a rotating ellipsoid in which circular target cells and axons were arranged around the upper and lower part, respectively (see Figure 1). The release rate of the regeneration inhibitors on the scar surface and the neurotrophic factors (NTFs) at the target and the size and the shape of the glial scars were controlled in the simulation, respectively. Concentration gradients of three types of diffusible molecules were tested in this model: (1) Type 1 factors, that is, attractive molecules that are released by the target tissues after nerve injury (such as neurotrophic factor-1 and NGF); (2) Type 2 factors, that is, chondroitin sulfate proteoglycans (CSPGs) that are not neutralized by the Chase-ABC on the surface of glial scar; and (3) Type 3 factors, that is, a variety of growth promoting molecules produced by the transplanted Schwann cells on the surface of glial scar (such as laminin, fibronectin, and neural cell adhesion molecules). Type 1 factors played a leading role in axonal regeneration. Types 2 and 3 factors exhibited balanced and coordinated effects. In addition, cross talk between Type 1 factors and Type 2/3 factors took place through signal transduction [30]. Concentrations of all three types of molecules were denoted as *ρ*_1_, *ρ*_2_, and *ρ*_3_. Coupled reaction-diffusion equations represented concentrations of these three types of molecules varying in space and time, in which they were key parameters coaffecting the growth rate and whether regenerating axons grew or stopped elongating. The “growth equation” of regenerating axons was designed using gradient parameters according to the cell chemotaxis principle. Using a mouse model of spinal cord transaction [4, 5, 17, 31], a boundary condition was established. Numerical simulations were performed using the three-dimensional LBM to determine the quantitative relationship between growth velocity of regenerating axons and concentrations of promoters and inhibitors in a deprived environment. This study provides a theoretical reference for designing the related experiments.

## 2. Materials and Methods

### 2.1. Evolution Equations for the Concentration of Promoting and Inhibiting Factors in Microenvironment

From the physical and mathematical point of view, three types of diffusible molecules after SCI, the same as nervous system in developmental stages, should be subjected to the first law of Fick [18, 20–22]. The equations are

(1) ∂ρ1∂t=D1∇2ρ1−k−1ρ1+∑j=1NTσ1δr−rjT,

(2) ∂ρ2∂t=D2∇2ρ2−k−2ρ2+∑j=1NAσ2ρ1δr−rkA,

(3) ∂ρ3∂t=D3∇2ρ3−k−3ρ3+∑j=1NAσ3ρ1δr−rkA.

Expressions (1)–(3) are multicomponent nonstationary reaction-diffusion equations with nonlinear coupling point sources, where ∇^2^ = ∂^2^/∂*x* ^2^ + ∂^2^/∂*y* ^2^ + ∂^2^/∂*z* ^2^ (the Laplace operator) and **r** = *x* **i** + *y* **j** + *z* **k** (*x*, *y*, and *z* are coordinates, and **i**, **j**, and **k** are unit vectors in Cartesian coordinate system). *ρ*_1_, *ρ*_2_, and *ρ*_3_, the function for diffusible molecules at position **r** (*μ*m) at time *t* (s), are the concentration of Types 1, 2, and 3 of diffusible molecules, respectively. *D*_1_, *D*_2_, and *D*_3_ are corresponding diffusion coefficients (constants, *μ*m^2^s^−1^). *k*_−1_, *k*_−2_, and *k*_−3_ are the linear attenuation coefficients (constants, s^−1^). All ∑s are treated as point sources, and *δ*(**r**) is the Dirac delta function. *N*_*T*_ and *N*_*A*_ are the number of the target cells and axons, respectively. **r**_*j*_^*T*^ (stabilizing) is the position of the *j*th target cell, and **r**_*k*_^*A*^ (growing with the time) is the position of the *k*th growth cone. Constant *σ*_1_ (*μ*Ms^−1^) is the release rate of Type 1 factors. *σ*_2_(*ρ*_1_) and *σ*_3_(*ρ*_1_) are the release rate of Type 2 and Type 3 factors, respectively. These two parameters were expressed as a function of *ρ*_1_ (usually nonlinear) in several specific forms to reflect their interactions with Type 1 factors. *σ*_20_ and *σ*_30_ are the basic release rates of Type 2 and Type 3 factors, respectively. Consider *σ*_2_ = *σ*_20_(1 − *R*_*L*_) and *σ*_3_ = *σ*_30_ *R*_*L*_, *R*_*L*_ = *ρ*_1_/(*K*_*d*_ + *ρ*_1_). *R*_*L*_, a dimensionless quantity, is the associativity formula of receptor-ligand. *R*_*L*_ and (1 − *R*_*L*_) reflect the competitive relationship of succeeding each other between enhancing and inhibitory factors. *K*_*d*_ is the dissociation constant.

### 2.2. Equations for Axonal Growth

The movement of an axonal growth cone is chemotactic and biased towards (attractive chemotactic) or away (repulsive chemotactic) from the chemical source [32, 33]. The attractive or repulsive action on a growth cone is referred to as a chemotactic force, which is proportional to the gradient of diffusible molecules from the chemical source. If a growth cone is regarded as a particle, the growth rate of the axon depends on the velocity of the particle. Since axonal growth is very slow (~0.01–0.05 *μ*ms^−1^) [32, 33], acceleration or inertia forces can be negligible. Therefore, the velocity of the growth cone is directly proportional to the chemotactic force:

(4) drkAdt=1μFkA,k=1,2,…,NA,FkA=∑i=13λipi,pi=∇ρiΔrkAρΣ,ρΣ=∑i=13ρi,  i=1,2,3,

where **r**_*k*_^*A*^( = *x*_*k*_^*A*^ **i** + *y*_*k*_^*A*^ **j** + *z*_*k*_^*A*^ **k**)  is the position (*μ*m) of the *k*th growth cone of axons at time *t* (s), *N*_*A*_ is the number of regenerative axons, *μ* is the viscosity coefficient (Pa·s), **F**_*k*_^*A*^( = *F*_*xk*_^*A*^ **i** + *F*_*yk*_^*A*^ **j** + *F*_*zk*_^*A*^ **k**) is the total chemotactic force (pN) from all three types of molecules acting on the *k*th growth cone at time *t* (s), *i* is the number of molecular types, and the variables and parameters with subscript *i* relate to the *i*th type of molecules. We defined a dimensionless vector **p**_*i*_ with proportionality constant *λ*_*i*_ to express the chemotactic force, in which *ρ*_*i*_ is the concentration of type *i* molecules in position **r**_*k*_^*A*^ at time *t*, ∇*ρ*_*i*_ is the gradient of *ρ*_*i*_  (∇ = **i**∂/∂*x* + **j**∂/∂*y* + **k**∂/∂*z*, the Hamilton operator), $\left|\Delta r_{k}^{A}\right|=\sqrt{\Delta x^{2}+\Delta y^{2}+\Delta z^{2}}$ is the scalar quantity of difference of **r**_*k*_^*A*^, and *ρ*_Σ_ is the sum of *ρ*_*i*_ over all three types of molecules. The scalar quantity of **p**_*i*_ can be simplified into Δ*ρ*_*i*_/*ρ*_Σ_, the relative difference of *ρ*_*i*_, and Δ*ρ*_*i*_ is the absolute difference of *ρ*_*i*_ across a distance |Δ**r**_*k*_^*A*^|. In practice, one can take |Δ**r**_*k*_^*A*^| ~ 20 *μ*m as an average diameter of growth cone. Therefore, a bridge between gradient and relative difference of concentration is established through **p**_*i*_ mathematically, and the corresponding proportionality constant *λ*_*i*_ is given a clear physical meaning whose dimension is equal to [force]/[length]. Using this model, the rate distortion of the growth cone is greatly reduced when the growth cone is close to the target cells. In addition, for one-dimensional single-component problems [34–36], **p**_*i*_ can be reduced to *p* = (∂*ρ*/∂*x*)·(Δ*x*/*ρ*) or *p* = Δ*ρ*/*ρ* which is consistent with the definition of gradient of concentration in some biophysical areas.

### 2.3. The Curved Surface Equation and Condition of Glial Scar Surface

Neural cell adhesion molecule L1 can be synthesized by Schwann cells [37], and this is considered to be one of the major reasons why axons can grow firmly attached to the surface of glial scars. In order to reflect this performance of Schwann cells, a constraint equation, the curved surface equation of glial scar surface, was included in the present model.

Due to the hypothesis that glial scar is simplified to a rotating ellipsoid, where the horizontal rotation radius $r=\sqrt{x^{2}+y^{2}}$ (*x*, *y*, and *z* are coordinates in Cartesian system), the lengths of horizontal half axle and vertical half axle are *r*_*a*_ and *r*_*z*_, respectively, and the sphere center coordinate is *O*(*x*_0_, *y*_0_, *z*_0_), and the constraint equation of scar surface can be expressed as

(5) $$\begin{array}{c} r=r_{a}\sqrt{1-\frac{{\left(z-z_{0}\right)}^{2}}{r_{z}^{2}}}. \end{array}$$

Thus, the movement of regenerating axons' growth cones is transformed into a particle motion along the surface of the rotation ellipsoid. The constraint relations for the velocity can be obtained by taking the time derivative of both sides of (5); we can get the constraint relations for the velocity:

(6) Vr=rarz2z0−zrVz,Vr=drdt,  Vz=dzdt,Vx=dxdt=Vrxr,Vy=dydt=Vryr,

where *V*_*z*_ is the drawing speed, a longitudinal component of velocity of an axon growing along the scar surface, and can be used to represent a growth rate of axons. Using growth equations (4), *V*_*z*_ in *k*th axon can be rewritten as

(7) Vzk=dzkAdt=1μFzkA,k=1,2,…,NA,FzkA=∑i=13λi∂ρi∂z·ΔzρΣ,ρΣ=∑i=13ρi.

The meanings of symbols in these equations can be found in Section 2.2.

### 2.4. Development of the Mathematical Model and Numerical Methods

This paper created a mathematical model which included (1)–(7). Based on the experiment for the SCI repair by transplanting Schwann cells [17], a numerical simulation method was used to explore the regenerative axons growing along the glial scar surface.

Equations (2) and (3) revealed that the source terms nonlinearly related to (4) through point **r**_*k*_^*A*^, and the point source was moving. So (1)–(4) were a set of coupled nonlinear partial differential equations which could only be solved numerically.

The solution of this model contained three steps and three methods: the first step, using the LBM [26–29] to solve for the concentration field of various factors determined by the reaction-diffusion equations (1)–(3); the second step, using the central difference method to solve for gradients of various factors surrounding the growth cone, and axon growth rates would be solved when the solution for the gradient was then inserted into (4); and, finally, using Euler's method to numerically integrate (4) and solving for the axonal growth path.

The simulation was implemented in two ways: one was to change the release rate of inhibitory factors, and the other was to change the scar diameter. Axonal growth rates were simulated and recorded in simulations. Whether regenerating axons could grow across or around the scar tissue to connect with the target cells was also tested.

In nerve cells, there were reliable data in the order of magnitude obtained from in vitro experiments as follows [34–36]: the width of the growth cone 10–20 *μ*m, the rate of axon growth 0.01–0.05 *μ*ms^−1^, the diffusion coefficient of NGF *D*_1_ ≈ 100 *μ*m^2^ s^−1^, and dissociation constant *K*_*d*_ ≈ 1 nM in which NGF bind with receptors of the growth cone membrane. In addition, the concentration range was at 0.01*K*_*d*_–10*K*_*d*_ and minimum relative concentration difference was 1% which would work on the growth cone. However, there were many data which had not been recognized, including point source release rates of NGF *σ*_1_, attenuation coefficient *k*_−1_, constant growth rate of axon *λ*_*i*_, and viscosity coefficient *μ*. For the above reason, the principle that the diffusion velocity of NGF $k_{-1}\sqrt{D_{1}/k_{-1}}$ should be greater than the growth cone velocity was used to calculate attenuation coefficient *k*_−1_. Then, using the numerical method, how much point source release rate was needed to generate a concentration range of 0.01*K*_*d*_–10*K*_*d*_ and a minimum relative concentration difference of 1% could be calculated. How much *λ*_*i*_/*μ* was needed to achieve an axon growth of 0.01–0.05 *μ*ms^−1^ in this concentration field could also be extrapolated. Parameters of Type 2 and Type 3 factors were given by the relative ratio of NGF.

## 3. Results and Discussion

### 3.1. Influence of Inhibitor Release Rate on Axonal Regeneration

In this section, the following hypothesis was tested through numerical simulation: although glial scar exists, it does not affect the growth of regenerating axons as long as the inhibitor level on the surface of the glial scar is below a threshold.

In the simulation, the size and shape of the glial scar were set to a fixed value and inhibitor release rates from their surface were varied. Growth rates of regenerating axons and the concentration of influencing factors near growth cones were recorded to quantitatively analyze the relationships between them. To simplify the process, a cubical compartment with a side length of *L* = 6720 *μ*m (see Figure 1) was considered. The central portion of the compartment was an ellipsoidal obstruction composed of glial scar (take spherical glial scar as an example; the radius is 0.26 × *L*). The equidistant distribution of small stars at the bottom of the sphere represented a cluster of axons regrown by the remnants of neurons (there were 12 branched axons). Tiny bubbles nearby the top of the sphere represented target cells that could release the NTFs. In all cases in this section, the time step size (d*t*) was one. Various values of parameters in (1)–(3) and (4) were the characteristic diffusion distance ($\sqrt{D_{1}/k_{-1}}=1000$ *μ*m) of chemoattractant molecules based mainly on NTFs and dissociation constant *K*_*d*_ = 1 nM. The relative values of diffusion coefficient of each factor were *D*_1_/*D*_2_ = *D*_1_/*D*_3_ = 10/3. The relative values of attenuation coefficient were *k*_−1_/*k*_−2_ = *k*_−1_/*k*_−3_ = 3 × 10^−3^. The ratios of the growth rate coefficient of the axonal growth cones were *λ*_1_/(−*λ*_2_) = *λ*_1_/*λ*_3_ = 1, *λ*_1_/*μ* = 1 *μ*ms^−1^. The ratio of the basic release rate of Type 1 factors was *σ*_1_ = 6 × 10^−3^ *μ*Ms^−1^. The ratios of the basic release rate of Type 2 and Type 3 factors (release ratio) were *η*_2_ = *σ*_20_/*σ*_1_, *η*_3_ = *σ*_30_/*σ*_1_, and *η*_3_ = 3%. The values of these parameter were chosen based on the previous work [34–36] and calculations discussed in Section 2.4.

**Figure 2:**
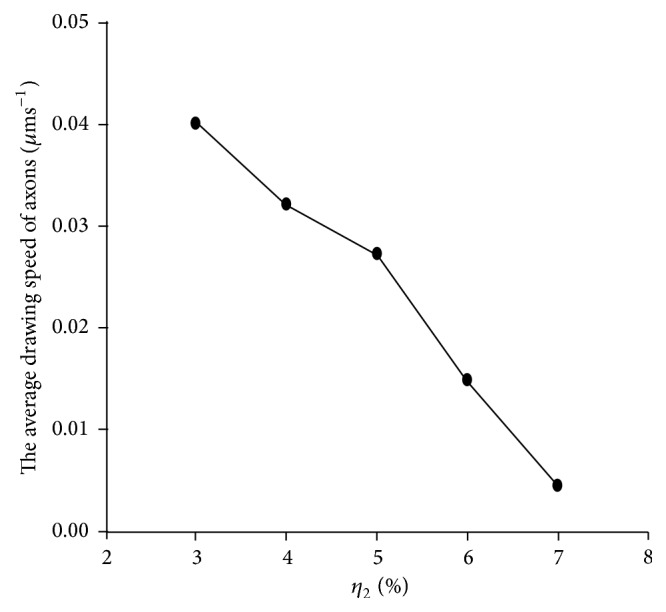
The trend of changes in the average drawing speed of axons with changes in the release rate of growth inhibitory factors (*η*_2_). The average drawing speed of axons decreases with increase in the value of *η*_2_.

From Figure 2 and Table 1, it is clear that the average drawing speed of axons (*V*_*zk*_ defined by (7)) decreased gradually with the increase of the release rate of growth inhibitory factors. When *η*_2_ was 3%, all axons were successfully connected to their target cells (see Figure 1(a)). So when *η*_2_ was less than 3%, inhibitory factor did not affect axonal regeneration along the glial scar. When *η*_2_ was at 4%, 5%, or 6%, the number of axons successfully connected to the target cell gradually reduced and the average drawing speed of axons slowed significantly (see Figure 1(b)). No axons could regenerate when *η*_2_ was equal to 7% (see Figure 1(c) and Table 1). These results showed that Type 2 factor was increased gradually and making a greater impact to hinder axonal regeneration with the increase value of *η*_2_ when diffusion coefficients, dissipation coefficients, and other parameters remained unchanged.

### 3.2. Influence of Size and Shape of Glial Scar on Axonal Regeneration

In this section, axon growth behavior along glial scars with different sizes and shapes was explored. For illustration purpose, five different glial scars (five cases), which were named number 1, number 2, number 3, number 4, and number 5, were used. Here, number 1 is horizontal half-axle (defined in Section 2.3) *r*_*a*1_ = 0.26 × *L* and vertical half-axle *r*_*z*1_ = 0.2 × *L*; number 2 is *r*_*a*2_ = 0.2 × *L* and *r*_*z*2_ = 0.26 × *L*; number 3 is *r*_*a*3_ = *r*_*z*3_ = 0.26 × *L*; number 4 is *r*_*a*4_ = 0.32 × *L* and *r*_*z*4_ = 0.26 × *L*; and number 5 is *r*_*a*5_ = 0.26 × *L* and *r*_*z*1_ = 0.32 × *L*. In all cases in this section, the ratios of the basic release rate of Type 2 and Type 3 to Type 1 factors (release ratio) were *η*_2_ = *σ*_20_/*σ*_1_ = *η*_3_ = *σ*_30_/*σ*_1_ = 3%.

**Figure 3:**
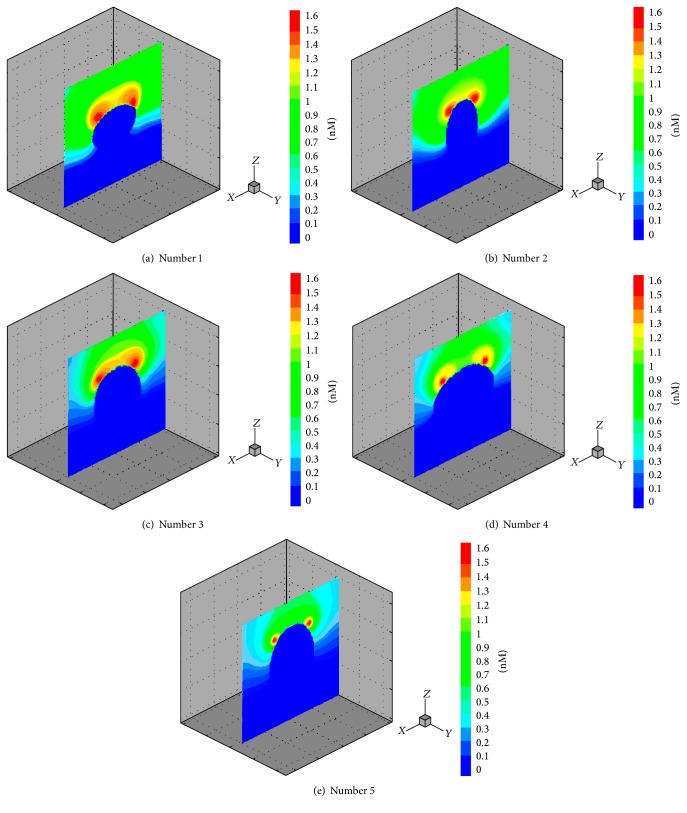
Longitudinal sections for three-dimensional concentration field of Type 1 factors. The concentration of the dark blue area equals zero and the red area represents the highest concentration. The target cells were located in two red circular domains nearby the top of glial scar. (a, c, e) The horizontal half axle of number 1, number 3, and number 5 remains constant at a value of 0.26 × *L* and their vertical half axle increases from 0.2 × *L* to 0.32 × *L* (increasing 0.06 × *L* each time). (b, c, d) The vertical half axle of number 2, number 3, and number 4 remains constant at a value of 0.26 × *L* and their transverse radius increases from 0.2 × *L* to 0.32 × *L* (increasing 0.06 × *L* each time).

**Figure 4:**
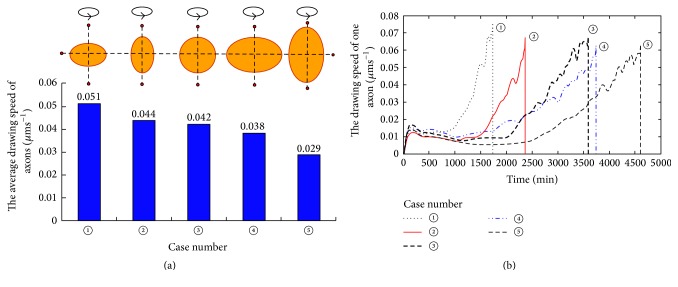
Axonal growth rate varied with size and shape of glial scars. (a) Histograms for the average drawing speed of the regenerating axons in different sizes and shapes of glial scars. (b) Time-history curves of the drawing speed of the regenerating axons. Data of one representative axon from each case was collected to plot the curve.

Figure 3 shows the longitudinal sections for three-dimensional concentration field of Type 1 factors when the axons connected with target cells. It is clear from Figures 3(a), 3(b), and 3(e) that the bypassing glial scar to spread remote areas by Type 1 factors becomes much more difficult with the increase of the vertical size of the glial scar. Figures 3(b), 3(c), and 3(d) show the result of a simulation in which the lateral diffusing capacity for diffusion factors has been weakened with the increase of the transverse size of the glial scar, and the concentration-diffusion is also weakened in the whole computational domain. In comparison to the impact of changes in the vertical radius, changes in the transverse size of glial scar have weaker effects on diffusion of these factors.  Figure 4 shows the axonal growth rates varying with size and shape of glial scars. It is clear from Figure 4(a) that the average drawing speed of regenerating axons became slower as the size of glial scar increased. The increase in transverse sizes of the glial scar did not cause significant reduction of axon growth rate. However, the increase in longitudinal size of the glial scar caused significant reduction of axon growth rate. For Figure 4(b), in these regenerating axons, one representative was selected and the time required for the representative axon to reach the target domain was calculated; the resulting curves also show that the longitudinal size of a glial scar had greater influence on the axon growth rate or the time required for an axon to reach its target domain.

## 4. Conclusions

This study focused on the physical point of view to explore the reasons for inhibition of CNS axon regeneration from the external microenvironment of the nerve cells. Based on the experiment for the SCI repair by Schwann cells transplantation and certain hypotheses, a new mathematical model was built. This model has two main control parameters: (1) the diameter of a glial scar and (2) the ratio of release rate of axon regeneration inhibitors on the scar surface to release rate of NTF from the target cells *η*_2_. From numerical calculations, simulations, and analyses, the model yielded the following outcomes: (1) Regenerating axons could successfully navigate across the glial scar and connect to their target cells with the support of Schwann cells and the guidance of NTFs concentration gradients of the target cells when *η*_2_ was less than 3%. (2) Changes in the longitudinal size of the glial scar had greater influence on the average rate of axon growth and the required time for axon to reach its target domain compared to the transverse size of the glial scar.

Implantation of Schwann cells and chondroitinase is now considered to be one of the most promising treatment strategies for SCI repair [31]. Existing experimental results show that the regenerating axons can grow along the glial scar to reach the target cells occasionally [17]. Simulation results in this study are in satisfactory agreement with existing experimental data in certain conditions. We would like to point out that, as the initial stage in developing this model, this study did not take into account the sprouting mechanisms after neuronal injury, the polymerization of the cytoskeletal protein within the growth cone of the regenerative axon, and other internal factors. Additional experimental data could be integrated to improve this model in the future.

The LBM for a three-dimensional numerical simulation was adopted in this study. This new method has several advantages, especially in dealing with complex boundaries, incorporating of microscopic interactions, and parallelization of the algorithm. First, there is no problem in principle if the impact factors are divided into more components and the glial scars with more complex shapes are taken into account. Although using only mathematical method does not discover and identify what is a promoting factor and inhibitory factor, it provides an important complement to experimental work to elucidate the ratio and distribution law of the impact factors. Second, this model provides a means of integrating data obtained from different experiments and laboratories. As illustrated in the last few sections of Results and Discussion, mathematical models can also have considerable predictive capabilities. After a model has been developed and carefully validated, it can be used to predict the inhibitory factor levels and the size of the glial scar that allow regenerating axons to successfully navigate across the glial scar. Ideally, experimental work and model development should be carried out in close association, using the mathematical model to guide the design and to evaluate experiments and using experimental results to improve the model development.

## Figures and Tables

**Table 1:** Comparison of the calculation results.

Release rate of growth inhibitory factors, *η*_2_ (%)	Average drawing speed of axons, *V*_*z* ave._ (*μ*ms^−1^)	The number of axons successfully connected to the target cells
3	0.0400	12
4	0.0320	10
5	0.0272	8
6	0.0148	3
7	0.0045	0
